# Supplementary material for: Modeling glioblastoma heterogeneity as a dynamic network of cell states
Source: Mol Syst Biol. 2021 Sep 16;17(9):e10105. doi: 10.15252/msb.202010105 (PMC8444284; doi:10.15252/msb.202010105)
Supplement: Supplementary file 5 — Source Data for Figure 3 [file MSB-17-e10105-s001.zip › Figure3A_sourcedata/GSEA_3065/nosigdb_state4.GseaPreranked.1622718430994/FELDMAN_2020_G1.html]

Details for gene set FELDMAN\_2020\_G1[GSEA]

|  || Dataset | state4 |
| Phenotype | NoPhenotypeAvailable |
| Upregulated in class | na\_neg |
| GeneSet | FELDMAN\_2020\_G1 |
| Enrichment Score (ES) | -0.8740101 |
| Normalized Enrichment Score (NES) | -3.3279803 |
| Nominal p-value | 0.0 |
| FDR q-value | 0.0 |
| FWER p-Value | 0.0 |
Table: GSEA Results Summary

  

Fig 1: Enrichment plot: FELDMAN\_2020\_G1      
 Profile of the Running ES Score & Positions of GeneSet Members on the Rank Ordered List

  

| PROBE | GENE SYMBOL | GENE\_TITLE | RANK IN GENE LIST | RANK METRIC SCORE | RUNNING ES | CORE ENRICHMENT || 1 | S100B |  |  | 6 | 0.630 | 0.0114 | No |
| 2 | PLP1 |  |  | 330 | 0.169 | -0.0381 | No |
| 3 | IGFBP5 |  |  | 901 | 0.096 | -0.1293 | No |
| 4 | PRCP |  |  | 1675 | 0.056 | -0.2546 | No |
| 5 | PDLIM7 |  |  | 2389 | 0.037 | -0.3704 | No |
| 6 | TUBA1B |  |  | 3658 | 0.007 | -0.5775 | No |
| 7 | CALM2 |  |  | 3844 | -0.001 | -0.6077 | No |
| 8 | MIAT |  |  | 4438 | -0.039 | -0.7039 | No |
| 9 | CMTM7 |  |  | 4735 | -0.063 | -0.7511 | No |
| 10 | CYCS |  |  | 4894 | -0.075 | -0.7754 | No |
| 11 | SCRG1 |  |  | 4970 | -0.080 | -0.7861 | No |
| 12 | LSM3 |  |  | 5075 | -0.089 | -0.8013 | No |
| 13 | RNASEH2A |  |  | 5241 | -0.105 | -0.8262 | No |
| 14 | PTN |  |  | 5261 | -0.107 | -0.8272 | No |
| 15 | ILF2 |  |  | 5331 | -0.112 | -0.8363 | No |
| 16 | H2AFV |  |  | 5381 | -0.117 | -0.8420 | No |
| 17 | RAD21 |  |  | 5496 | -0.131 | -0.8581 | No |
| 18 | MAP3K7CL |  |  | 5565 | -0.138 | -0.8665 | No |
| 19 | BUB3 |  |  | 5583 | -0.141 | -0.8665 | No |
| 20 | HMGB3 |  |  | 5584 | -0.142 | -0.8637 | No |
| 21 | CENPW |  |  | 5585 | -0.142 | -0.8609 | No |
| 22 | PSIP1 |  |  | 5590 | -0.143 | -0.8588 | No |
| 23 | DLG1 |  |  | 5592 | -0.144 | -0.8561 | No |
| 24 | KNSTRN |  |  | 5610 | -0.146 | -0.8560 | No |
| 25 | CCDC34 |  |  | 5647 | -0.152 | -0.8589 | No |
| 26 | NUCKS1 |  |  | 5724 | -0.166 | -0.8681 | No |
| 27 | EXOSC8 |  |  | 5751 | -0.173 | -0.8689 | No |
| 28 | MALAT1 |  |  | 5783 | -0.179 | -0.8705 | Yes |
| 29 | SIVA1 |  |  | 5791 | -0.181 | -0.8681 | Yes |
| 30 | HAT1 |  |  | 5803 | -0.184 | -0.8663 | Yes |
| 31 | MIS18A |  |  | 5816 | -0.187 | -0.8645 | Yes |
| 32 | CMC2 |  |  | 5834 | -0.191 | -0.8636 | Yes |
| 33 | PRC1 |  |  | 5835 | -0.192 | -0.8598 | Yes |
| 34 | PLK1 |  |  | 5836 | -0.192 | -0.8560 | Yes |
| 35 | PTMA |  |  | 5852 | -0.195 | -0.8546 | Yes |
| 36 | HIST1H1E |  |  | 5865 | -0.198 | -0.8527 | Yes |
| 37 | CKAP2 |  |  | 5879 | -0.201 | -0.8509 | Yes |
| 38 | ANP32B |  |  | 5890 | -0.204 | -0.8485 | Yes |
| 39 | C12orf75 |  |  | 5896 | -0.206 | -0.8453 | Yes |
| 40 | SNRPB |  |  | 5908 | -0.209 | -0.8430 | Yes |
| 41 | SNRPD1 |  |  | 5911 | -0.210 | -0.8392 | Yes |
| 42 | PTGES3 |  |  | 5912 | -0.210 | -0.8350 | Yes |
| 43 | LSM5 |  |  | 5932 | -0.216 | -0.8339 | Yes |
| 44 | RANBP1 |  |  | 5939 | -0.218 | -0.8306 | Yes |
| 45 | CENPA |  |  | 5960 | -0.226 | -0.8294 | Yes |
| 46 | TMEM106C |  |  | 5979 | -0.230 | -0.8279 | Yes |
| 47 | TROAP |  |  | 5983 | -0.232 | -0.8238 | Yes |
| 48 | USP1 |  |  | 5986 | -0.233 | -0.8195 | Yes |
| 49 | MZT1 |  |  | 5989 | -0.235 | -0.8152 | Yes |
| 50 | RAN |  |  | 5990 | -0.235 | -0.8106 | Yes |
| 51 | STMN1 |  |  | 5993 | -0.236 | -0.8063 | Yes |
| 52 | RPA3 |  |  | 6014 | -0.244 | -0.8048 | Yes |
| 53 | AURKA |  |  | 6026 | -0.248 | -0.8017 | Yes |
| 54 | HNRNPDL |  |  | 6028 | -0.251 | -0.7969 | Yes |
| 55 | HNRNPA2B1 |  |  | 6029 | -0.251 | -0.7920 | Yes |
| 56 | DEPDC1 |  |  | 6030 | -0.251 | -0.7870 | Yes |
| 57 | VRK1 |  |  | 6034 | -0.256 | -0.7825 | Yes |
| 58 | FEN1 |  |  | 6035 | -0.256 | -0.7775 | Yes |
| 59 | DNAJC9 |  |  | 6037 | -0.260 | -0.7725 | Yes |
| 60 | HMGB1 |  |  | 6047 | -0.267 | -0.7687 | Yes |
| 61 | HSP90AA1 |  |  | 6062 | -0.275 | -0.7656 | Yes |
| 62 | SPC25 |  |  | 6072 | -0.279 | -0.7616 | Yes |
| 63 | CEP55 |  |  | 6076 | -0.281 | -0.7565 | Yes |
| 64 | HIST1H1C |  |  | 6077 | -0.284 | -0.7510 | Yes |
| 65 | GMNN |  |  | 6080 | -0.285 | -0.7457 | Yes |
| 66 | FBXO5 |  |  | 6084 | -0.287 | -0.7405 | Yes |
| 67 | KIF22 |  |  | 6089 | -0.290 | -0.7355 | Yes |
| 68 | CENPN |  |  | 6090 | -0.291 | -0.7298 | Yes |
| 69 | DEK |  |  | 6092 | -0.292 | -0.7242 | Yes |
| 70 | NUF2 |  |  | 6093 | -0.293 | -0.7184 | Yes |
| 71 | CCNA2 |  |  | 6100 | -0.297 | -0.7136 | Yes |
| 72 | DUT |  |  | 6107 | -0.303 | -0.7086 | Yes |
| 73 | CKAP2L |  |  | 6108 | -0.303 | -0.7026 | Yes |
| 74 | PHF19 |  |  | 6129 | -0.322 | -0.6996 | Yes |
| 75 | LMNB1 |  |  | 6134 | -0.325 | -0.6939 | Yes |
| 76 | CDCA2 |  |  | 6135 | -0.326 | -0.6875 | Yes |
| 77 | CENPH |  |  | 6136 | -0.327 | -0.6810 | Yes |
| 78 | DDX39A |  |  | 6137 | -0.328 | -0.6746 | Yes |
| 79 | CCNB2 |  |  | 6140 | -0.329 | -0.6684 | Yes |
| 80 | NDC80 |  |  | 6142 | -0.330 | -0.6621 | Yes |
| 81 | AURKB |  |  | 6143 | -0.332 | -0.6556 | Yes |
| 82 | TK1 |  |  | 6144 | -0.334 | -0.6490 | Yes |
| 83 | ARL6IP1 |  |  | 6145 | -0.336 | -0.6424 | Yes |
| 84 | NUDT1 |  |  | 6146 | -0.338 | -0.6357 | Yes |
| 85 | RRM1 |  |  | 6147 | -0.338 | -0.6291 | Yes |
| 86 | SRSF7 |  |  | 6150 | -0.343 | -0.6227 | Yes |
| 87 | CDC20 |  |  | 6154 | -0.344 | -0.6164 | Yes |
| 88 | DLGAP5 |  |  | 6156 | -0.345 | -0.6098 | Yes |
| 89 | CDKN3 |  |  | 6160 | -0.349 | -0.6034 | Yes |
| 90 | KIF23 |  |  | 6162 | -0.351 | -0.5967 | Yes |
| 91 | HMGN2 |  |  | 6163 | -0.351 | -0.5898 | Yes |
| 92 | KIF4A |  |  | 6164 | -0.355 | -0.5828 | Yes |
| 93 | CDCA3 |  |  | 6165 | -0.355 | -0.5758 | Yes |
| 94 | ANP32E |  |  | 6168 | -0.357 | -0.5691 | Yes |
| 95 | CENPK |  |  | 6169 | -0.363 | -0.5620 | Yes |
| 96 | KIF20B |  |  | 6172 | -0.373 | -0.5550 | Yes |
| 97 | PCNA |  |  | 6173 | -0.374 | -0.5476 | Yes |
| 98 | DTYMK |  |  | 6177 | -0.381 | -0.5406 | Yes |
| 99 | CENPM |  |  | 6179 | -0.386 | -0.5332 | Yes |
| 100 | DBF4 |  |  | 6180 | -0.387 | -0.5256 | Yes |
| 101 | DNMT1 |  |  | 6182 | -0.396 | -0.5180 | Yes |
| 102 | IGFBP3 |  |  | 6184 | -0.397 | -0.5103 | Yes |
| 103 | GINS2 |  |  | 6194 | -0.420 | -0.5036 | Yes |
| 104 | ZWINT |  |  | 6195 | -0.425 | -0.4952 | Yes |
| 105 | TUBB4B |  |  | 6196 | -0.431 | -0.4867 | Yes |
| 106 | RRM2 |  |  | 6199 | -0.438 | -0.4784 | Yes |
| 107 | DHFR |  |  | 6200 | -0.441 | -0.4698 | Yes |
| 108 | ESCO2 |  |  | 6206 | -0.463 | -0.4615 | Yes |
| 109 | PBK |  |  | 6207 | -0.465 | -0.4523 | Yes |
| 110 | BIRC5 |  |  | 6214 | -0.484 | -0.4438 | Yes |
| 111 | ANLN |  |  | 6215 | -0.484 | -0.4343 | Yes |
| 112 | GTSE1 |  |  | 6217 | -0.490 | -0.4248 | Yes |
| 113 | CLSPN |  |  | 6218 | -0.490 | -0.4152 | Yes |
| 114 | SMC4 |  |  | 6219 | -0.490 | -0.4055 | Yes |
| 115 | TYMS |  |  | 6222 | -0.501 | -0.3960 | Yes |
| 116 | NUSAP1 |  |  | 6223 | -0.507 | -0.3860 | Yes |
| 117 | CENPE |  |  | 6226 | -0.511 | -0.3763 | Yes |
| 118 | NCAPG |  |  | 6227 | -0.513 | -0.3662 | Yes |
| 119 | SMC2 |  |  | 6229 | -0.518 | -0.3562 | Yes |
| 120 | GGH |  |  | 6232 | -0.522 | -0.3463 | Yes |
| 121 | TMPO |  |  | 6233 | -0.522 | -0.3360 | Yes |
| 122 | CKS1B |  |  | 6234 | -0.523 | -0.3257 | Yes |
| 123 | KPNA2 |  |  | 6236 | -0.543 | -0.3152 | Yes |
| 124 | H2AFX |  |  | 6237 | -0.576 | -0.3039 | Yes |
| 125 | ORC6 |  |  | 6238 | -0.585 | -0.2924 | Yes |
| 126 | CKS2 |  |  | 6239 | -0.585 | -0.2809 | Yes |
| 127 | UBE2T |  |  | 6240 | -0.593 | -0.2692 | Yes |
| 128 | CCNB1 |  |  | 6242 | -0.606 | -0.2575 | Yes |
| 129 | ATAD2 |  |  | 6243 | -0.607 | -0.2455 | Yes |
| 130 | UBE2S |  |  | 6244 | -0.617 | -0.2334 | Yes |
| 131 | PTTG1 |  |  | 6245 | -0.622 | -0.2212 | Yes |
| 132 | CDK1 |  |  | 6246 | -0.627 | -0.2088 | Yes |
| 133 | MAD2L1 |  |  | 6247 | -0.628 | -0.1965 | Yes |
| 134 | CENPU |  |  | 6248 | -0.633 | -0.1840 | Yes |
| 135 | HIST1H1B |  |  | 6249 | -0.646 | -0.1713 | Yes |
| 136 | H2AFZ |  |  | 6250 | -0.651 | -0.1586 | Yes |
| 137 | MCM7 |  |  | 6252 | -0.665 | -0.1456 | Yes |
| 138 | TPX2 |  |  | 6253 | -0.667 | -0.1325 | Yes |
| 139 | ASPM |  |  | 6254 | -0.680 | -0.1192 | Yes |
| 140 | UBE2C |  |  | 6255 | -0.750 | -0.1044 | Yes |
| 141 | SCG2 |  |  | 6257 | -0.758 | -0.0897 | Yes |
| 142 | HMGB2 |  |  | 6258 | -0.766 | -0.0746 | Yes |
| 143 | TOP2A |  |  | 6260 | -0.814 | -0.0587 | Yes |
| 144 | MKI67 |  |  | 6261 | -0.882 | -0.0414 | Yes |
| 145 | HIST1H4C |  |  | 6262 | -1.038 | -0.0210 | Yes |
| 146 | CENPF |  |  | 6263 | -1.068 | 0.0000 | Yes |
Table: GSEA details [plain text format]

  

Fig 2: FELDMAN\_2020\_G1: Random ES distribution      
 Gene set null distribution of ES for **FELDMAN\_2020\_G1**
